# Evidence-based veterinary medicine and contextualised care

**DOI:** 10.18849/ve.v11i3.754

**Published:** 2026-07-06

**Authors:** Peter Cockcroft

**Keywords:** EVIDENCE-BASED VETERINARY MEDICINE, CONTEXTUALISED CARE

## Abstract

The welfare and the quality of life of the patient are central to the practice of veterinary medicine. With this in mind there is a growing number of treatment options available and factors related to the patient, the owner/carer, the type of consultation, the profession, the practice, the wider society, and sustainability both environmental and financial that should be considered when making a decision about the management of a patient. The similar terms contextualised care and spectrum of care have been used to describe these considerations.

Decision making in the practice of contextualised care/spectrum of care, whichever options are under consideration, should be informed by the best current evidence from scientific research. The primary role of the *Veterinary Evidence* journal is to provide the scientific evidence from published research in a form that can be interpreted by veterinary professionals and which can used to inform their decision making.

## Contextualised care and the spectrum of care

The welfare and the quality of life of the patient are central to the practice of veterinary medicine. Advances in technology, an increase in the range of diagnostic tests available, and the expansion of referral practice in the profession have focused attention on the range of different patient management options available and additional factors that need to be taken into consideration (Englar, 2023a, 2023b; Skipper et al., 2024; Fredesco, 2025). The terms contextualised care (Skipper et al., 2024) and spectrum of care (Brown et al., 2021; Fingland, 2021) have been used to describe these considerations.

Contextualised veterinary care takes into account the aims, knowledge, experiences, and circumstances of individual animal caregivers and veterinary professionals, acknowledging the wider contexts of each clinical encounter, to deliver the most appropriate welfare focused care for every animal (Skipper et al., 2024). Factors related to the patient, owner/carer, type of consultation, profession, wider society, and sustainability should be considered (RCVS Knowledge, 2025).

The spectrum of care similarly refers to the wide range of care options veterinarians can provide and tailoring the care options based on contextual factors, such as client goals, abilities, and resources, as well as patient, veterinarian, and practice factors, while considering available evidence (Englar, 2023a, 2023b). These factors have been described in detail (Englar, 2023a, 2023b).

The *Veterinary Evidence* journal has recently published the findings of a survey investigating barriers to the delivery of contextualised care in UK small animal veterinary practice (Everitt et al., 2026). In parallel, RCVS Knowledge has developed a roadmap which describes the support that is needed to implement contextualised care (RCVS Knowledge, 2025).

## Role of the journal in supporting contextual care/spectrum of care

The primary purpose of the *Veterinary Evidence* journal is to publish Knowledge Summaries that critically appraise scientific research related to important clinical questions which have an impact on patient care. Critical appraisal enables the strength of evidence provided by a study to be defined and the validity of the reported outcomes to be established. This information supports the practice of evidence-based veterinary medicine (EBVM) by veterinarians and veterinary nurses, and facilitates the translation of research into clinical practice to enhance the quality of care provided to animals.

There are 5 steps described in the practice of EBVM (adapted from Sackett, 1997):

1. Defining a clinically relevant question
2. Searching for the best available evidence
3. Critically appraising the evidence
4. Applying the evidence
5. Evaluating the performance

**Figure 1:**
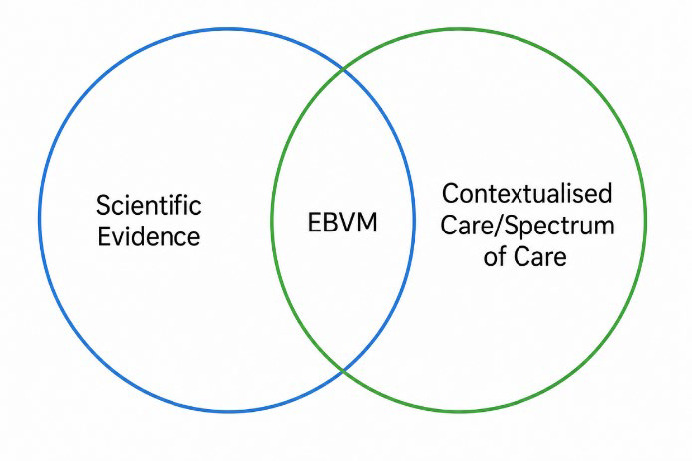
Venn diagram illustrating the relationship between scientific evidence, evidence-based veterinary medicine and contextualised care/spectrum of care.

A Knowledge Summary performs steps 1, 2, and 3. The responsibility for step 4, applying the evidence in clinical decision-making, and step 5, evaluating the performance, rests with the veterinarian. The relationship between scientific evidence, evidence-based veterinary medicine and contextualised care/spectrum of care is shown in Fig. 1.

Decision making in the practice of contextualised care/spectrum of care, whichever options are under consideration, should be informed by the best current evidence from scientific research.

The role of the *Veterinary Evidence* journal is to provide the scientific evidence from published research in a form that can be interpreted by veterinary professionals and inform their decision making. Contextualised care and spectrum of care prompt us to consider what we know to be true and why, so that we actively re-examine evidence-based options, and the way we deliver those options to clients (Englar, 2023a).

## ORCiD

Peter Cockcroft: 0000 0003 0090 0706

## Disclaimer

Any opinions expressed in articles and other publication types published in Veterinary Evidence are the author's own and do not necessarily reflect the view of the RCVS Knowledge. Veterinary Evidence is a resource to help inform, and the content herein should not override the responsibility of the practitioner. Practitioners should also consider factors such as individual clinical expertise and judgement along with patient’s circumstances and owners’ values. Authors are responsible for the accuracy of the content. While the Editor and Publisher believe that all content herein are in accord with current recommendations and practice at the time of publication, they accept no legal responsibility for any errors or omissions, and make no warranty, express or implied, with respect to material contained within. For further information please refer to our Terms of Use.
